# Shorter sleep time relates to lower human defensin 5 secretion and compositional disturbance of the intestinal microbiota accompanied by decreased short-chain fatty acid production

**DOI:** 10.1080/19490976.2023.2190306

**Published:** 2023-03-21

**Authors:** Yu Shimizu, Ryodai Yamamura, Yuki Yokoi, Tokiyoshi Ayabe, Shigekazu Ukawa, Koshi Nakamura, Emiko Okada, Akihiro Imae, Takafumi Nakagawa, Akiko Tamakoshi, Kiminori Nakamura

**Affiliations:** a Department of Cell Biological Science, Faculty of Advanced Life Science, Hokkaido University, Hokkaido, Japan; b Division of Biomedical Oncology, Institute for Genetic Medicine, Hokkaido University, Hokkaido, Japan; c Department of Social Welfare Science and Clinical Psychology, Osaka Metropolitan University Graduate School of Human Life and Ecology, Osaka, Japan; d Department of Public Health and Hygiene, Graduate School of Medicine, University of the Ryukyus, Okinawa, Japan; e Department of Nutritional Epidemiology and Shokuiku, National Institute of Biomedical Innovation, Health and Nutrition, Tokyo, Japan; f Suttu Municipal Clinic, Hokkaido, Japan; g The Centre for Family Medicine, Hokkaido, Japan; h Department of Public Health, Faculty of Medicine, Hokkaido University, Hokkaido, Japan

**Keywords:** Short sleep, α-defensin, Paneth cell, Intestinal microbiota, Short-chain fatty acid, Sleep disorder

## Abstract

Sleep is essential for our health. Short sleep is known to increase disease risks via imbalance of intestinal microbiota, dysbiosis. However, mechanisms by which short sleep induces dysbiosis remain unknown. Small intestinal Paneth cell regulates the intestinal microbiota by secreting antimicrobial peptides including α-defensin, human defensin 5 (HD5). Disruption of circadian rhythm mediating sleep-wake cycle induces Paneth cell failure. We aim to clarify effects of short sleep on HD5 secretion and the intestinal microbiota. Fecal samples and self-reported sleep time were obtained from 35 healthy middle-aged Japanese (41 to 60-year-old). Shorter sleep time was associated with lower fecal HD5 concentration (*r* = 0.354, *p* = 0.037), lower centered log ratio (CLR)-transformed abundance of short-chain fatty acid (SCFA) producers in the intestinal microbiota such as *[Ruminococcus] gnavus group* (*r* = 0.504, *p* = 0.002) and *Butyricicoccus* (*r* = 0.484, *p* = 0.003), and lower fecal SCFA concentration. Furthermore, fecal HD5 positively correlated with the abundance of these genera and SCFA concentration. These findings suggest that short sleep relates to disturbance of the intestinal microbiota via decreased HD5 secretion.

## Introduction

Sleep is an evolutionarily conserved phenomenon involving in essential roles in maintaining life by regulating various physiological functions such as recovery from physical and mental fatigue,^1^ growth and repair of muscles,^2^ enhancement of immune functions,^3^ clearance of waste products in the brain,^4^ and memory consolidation.^5^ Short sleep has been known to relate to increased risk of many diseases such as cardiovascular diseases, cerebrovascular diseases, hypertension, cancer, diabetes, and depression^6^ and further higher mortality rate,^7^ indicating that short sleep negatively affects our physical and mental health. According to an international survey conducted by National Sleep Foundation, 66% of Japanese, 53% of American, and 39% of British people sleep less than 7 h on workdays, and even more than half in each country feel that they got less sleep than needed.^8^ Thus, short sleep has become a global problem in public health. In addition, it has been reported that sleep time in Japanese among various age-groups showed U-shaped distribution indicating 40s and 50s get shortest sleep time^9^ and in Finland, people in the age-group of 35–54 years showed highest decrease of sleep time among three age-groups (18–34 years, 35–54 years, and over 55 years) in the longitudinal study^10^, suggesting that middle-aged people are at particularly high risk of short sleep among all age-groups.

The human intestinal microbiota consists of approximately 40 trillion bacteria^11^ and is estimated to possess more than 10 million genes.^12^ The intestinal microbiota contributes to many aspects of host physiological functions including energy intake, vitamin synthesis, bile acid metabolism, immune cell differentiation, and nervous system development.^13^ On the other hand, an imbalance of the intestinal microbiota, dysbiosis is involved in various diseases such as inflammatory bowel diseases, hypertension, arteriosclerosis, cancer, diabetes, autism, and depression.^14,15^ Recently, it has been reported that the intestinal microbiota composition relates to sleep quality^16^ and diversity of the intestinal microbiota positively correlates with sleep efficacy in several cross-sectional studies,^17^ whereas short-term experimental sleep restriction does not induce significant compositional changes of the intestinal microbiota.^18^ In addition, it is reported that healthy subjects whose daily sleep time was restricted to 4 h show increased Bacillota/Bacteroidota ratio (formerly known as Firmicutes/Bacteroidetes ratio) in the intestinal microbiota and increased insulin resistance compared to control subjects who got 8 h of sleep.^19^ Furthermore, in sleep fragmentation-treated mice exposed to intermittent contact stimulation, increased food intake, increased insulin resistance, and inflammation of adipose tissue as well as the whole body, along with compositional change of the intestinal microbiota were observed. These pathologies were reproduced in germ-free mice with normal sleep by transplantation of the intestinal microbiota of the sleep fragmentation-treated mice.^20^ These previous studies suggest that dysbiosis induced by sleep deprivation is involved in higher risk of various diseases. However, the mechanism that short sleep affects the intestinal microbiota composition remains unknown.

Intestinal epithelial cells are the first line of defense against microorganisms and play an important role in regulation of the intestinal microbiota composition.^21^ Paneth cells, a lineage of small intestinal epithelial cells residing at the base of the crypt, express antimicrobial peptides, α-defensins termed cryptdins (Crps) in mice^22^ and human defensin (HD) 5 and 6 in humans in their intracellular granules.^23,24^ Paneth cells contribute to innate enteric immunity by secreting the granules rich in α-defensins into the intestinal lumen in response to bacteria,^25,26^ food, and metabolic components.^27^ In addition, Paneth cell α-defensins secreted into the small intestinal lumen reach the large intestine and even in feces,^28^ contributing to regulation of the intestinal microbiota composition. α-Defensins selectively kill pathogenic bacteria whereas show no or minimal bactericidal activities against commensal bacteria.^29^ In addition, HD5 transgenic mice show different small intestinal microbiota composition compared to wild-type mice.^30^ Paneth cells further support regeneration and differentiation of the small intestinal epithelial cells by constituting a stem cell niche with adjacent intestinal epithelial stem cells.^31–33^ Recent evidences showed that structural abnormalities or decreased levels of Paneth cell α-defensins are involved in the pathologies of dysbiosis-related diseases such as Crohn’s disease,^34–36^ obesity,^37^ graft-versus-host disease (GVHD),^38,39^ and alcoholic steatohepatitis.^40^ They also are involved in compositional and metabolic disturbance of the intestinal microbiota associated with psychological stress^41^ and aging,^42^ suggesting that α-defensins play an important role in our health and disease via modulating the intestinal microbiota.

Sleep-wake cycle is generated in part by oscillatory expression of clock genes such as *Clock, Bmal1, Period* (*Per*), and *Cryptochrome* in suprachiasmatic nucleus (SCN) of hypothalamus.^43^ Also, timed sleep restriction-treated mice that mimic shiftwork in early morning or midnight show disturbed expression cycle of clock genes in SCN and liver,^44^ suggesting that inappropriate sleep induces disruption of circadian rhythm. Clock genes are widely expressed not only in central nervous system but also in various peripheral tissues.^45^ In intestine, clock genes are strongly expressed in the myenteric plexus and epithelial cells.^46,47^ Recently, involvement of circadian rhythm in the regulation of Paneth cell functions has been shown. Paneth cells synchronize cell division cycle of the intestinal epithelial stem cells and progenitor cells by periodically supplying Wnt, and functional knockdown of *Bmal1* or *Per1/2* impairs the Wnt secretion cycle of Paneth cells.^48^ It is also reported that the number of Paneth cells decreases in *Per1/2* mutant mice,^49^ and the expression level of Crps shows circadian oscillation in wild-type mice.^50^ Thus, short sleep is suggested to induce abnormalities in α-defensin secretion from Paneth cells through disruption of circadian rhythm, leading to compositional and functional impairment of the intestinal microbiota resulting in increased risk of disease. However, whether sleep affects the secretion of Paneth cell α-defensins remains unknown.

Here we aim to elucidate the effect of sleep time on the secretory levels of HD5 and composition and function of the intestinal microbiota in middle-aged people by conducting and analyzing the Dynamics of Lifestyle and Neighborhoods Community on Health Study (DOSANCO Health study), a community-based study of residents in Hokkaido, Japan.^51^

## Results

### Shorter sleep time correlates with lower HD5 secretion

**Figure 1.**
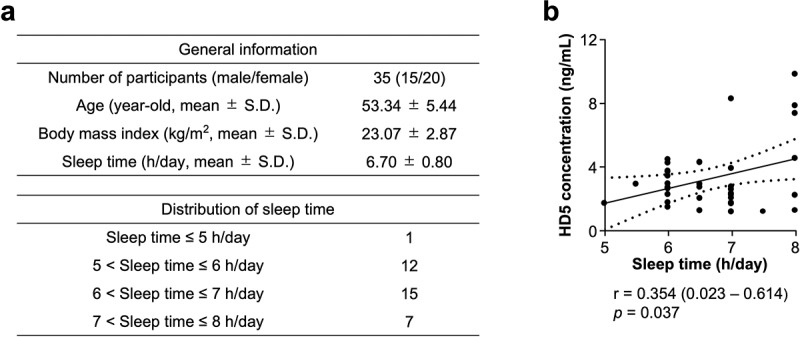
Shorter sleep time is associated with lower HD5 concentration.

To analyze the relationship between sleep time and HD5 secretion, correlation analysis between individual sleep time based on self-reported questionnaire and fecal HD5 concentration measured by sandwich ELISA was conducted (Figure 1a). Individual sleep time in all participants positively correlated with fecal HD5 concentration (Figure 1b), indicating that shorter sleep time is associated with lower secretory amount of HD5 into the intestinal lumen.

(a) General information and distribution of sleep time in participants of this study. (b) Correlation analysis between sleep time and fecal HD5 concentration in all participants. Statistical significance was evaluated by Pearson’s correlation coefficient test. Values in parentheses and dashed lines in the plot represent the range of 95% confidence interval.

### Shorter sleep time is associated with compositional disturbance of the intestinal microbiota accompanied by lower SCFA production

**Figure 2.**
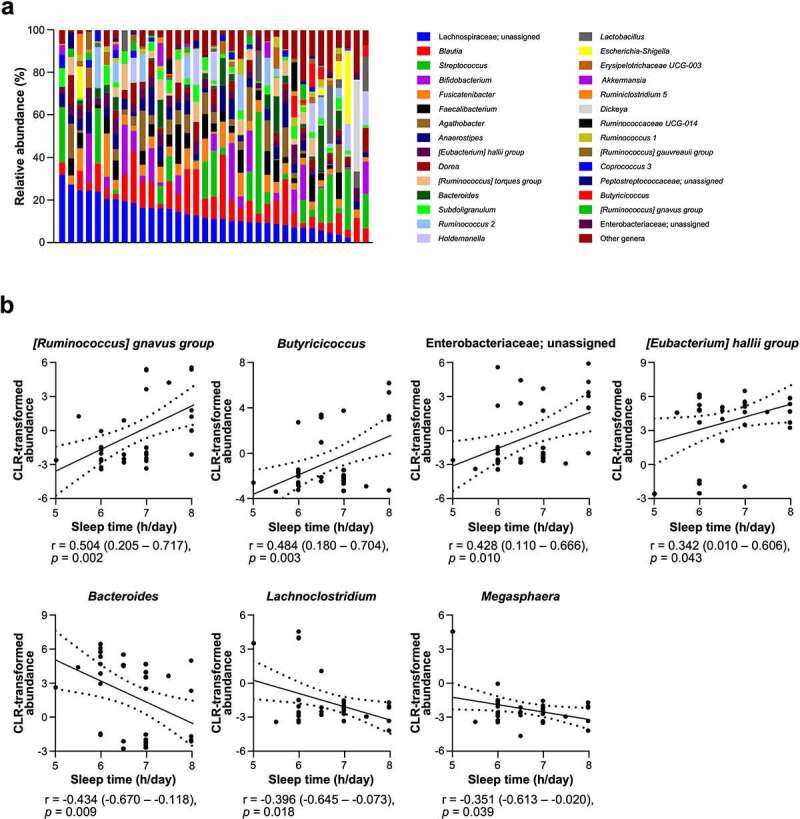
Sleep time is associated with occupancy of several genera in the intestinal microbiota .

**Figure 3.**
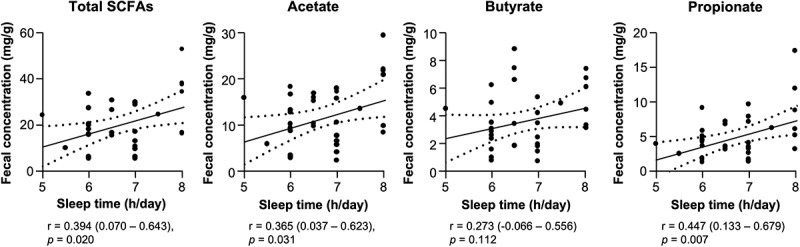
Shorter sleep time is associated with lower fecal SCFA concentration.

Next, to analyze whether short sleep effects on the intestinal microbiota, correlation analysis between sleep time and relative abundance of each genus in the intestinal microbiota by fecal 16S rDNA sequencing was conducted (Figure 2a). Sleep time positively correlated with centered log ratio (CLR)-transformed abundance of *[Ruminococcus] gnavus group*, *Butyricicoccus*, Enterobacteriaceae; unassigned and *[Eubacterium] hallii group*, and negatively correlated with *Bacteroides*, *Lachnoclostridium*, and *Megasphaera*, indicating shorter sleep time relates to compositional disturbance of the intestinal microbiota (Figure 2b). Because *[Ruminococcus] gnavus group* and *Butyricicoccus* positively correlated with sleep time are known as SCFA producers,^52,53^ we further determined whether short sleep relates to decreased SCFA production by the intestinal microbiota. Relationship between sleep time and fecal concentrations of acetate, butyrate, propionate, and total SCFAs (sum of these SCFAs) was analyzed (Figure 3). Sleep time positively correlated with fecal concentrations of total SCFAs, acetate, and propionate. These results indicate that shorter sleep time is associated with lower SCFA production.

(a) Intestinal microbiota composition of each participant at the genus level. Participants were sorted by relative abundance of Lachnospiraceae; unassigned in descending order. Genera which average of relative abundance in all participants was below 0.5% were integrated as “Other genera”. (b) Correlation analysis between sleep time and centered log ratio (CLR)-transformed abundance of each genus. Statistical significance was evaluated by Pearson’s correlation coefficient test. Values in parentheses and dashed lines in the plots represent the range of 95% confidence interval.

Correlation analysis between sleep time and fecal concentration of total and each SCFA. Statistical significance was evaluated by Pearson’s correlation coefficient test. Values in parentheses and dashed lines in the plots represent the range of 95% confidence interval.

### Lower HD5 secretion along with short sleep relates to compositional disturbance and lower SCFA production in the intestinal microbiota

Finally, correlation analysis between individual HD5 secretion and the intestinal microbiota composition or SCFA production was conducted (Table 1). Fecal HD5 concentration was positively correlated with CLR-transformed abundance of *[Ruminococcus] gnavus group*, *Butyricicoccus* and Enterobacteriaceae; unassigned which are genera showing positive correlation between sleep time, and negatively correlated with *Bacteroides* showing negative correlation between sleep time. HD5 concentration also positively correlated with fecal concentrations of total SCFAs, acetate, and propionate. These results indicate that lower HD5 secretion along with short sleep relates to compositional disturbance of the intestinal microbiota accompanied by lower SCFA production.

**Table 1.** Correlation analysis among fecal HD5 concentration, CLR-transformed abundance of sleep-related genera, and fecal SCFA concentration.

	vs Fecal HD5 concentration		
CLR-transformed abundance	r	95% CI	*p* value
*[Ruminococcus] gnavus group*	**0.416**	0.096 − 0.658	**0.013**
*Butyricicocccus*	**0.412**	0.091 − 0.655	**0.014**
Enterobacteriaceae; unassigned	**0.358**	0.028 − 0.617	**0.035**
*[Eubacterium] hallii group*	0.188	−0.155 − 0.491	0.279
*Bacteroides*	**−0.350**	−0.612 − −0.018	**0.040**
*Lachnoclostridium*	0.073	−0.267 − 0.397	0.676
*Megaspaera*	0.056	−0.283 − 0.382	0.751
Fecal SCFA concentration	r	95% CI	*p* value
Total SCFAs	**0.354**	0.024 − 0.615	**0.037**
Acetate	**0.351**	0.020 − 0.613	**0.039**
Butyrate	0.238	−0.104 − 0.529	0.169
Propionate	**0.364**	0.035 − 0.622	**0.032**

Statistical significance was evaluated by Pearson’s correlation coefficient test. **Bold font** indicates statistically significant. CI: confidence interval.

## Discussion

To analyze relationships among sleep time, fecal level of Paneth cell α-defensin, and the intestinal microbiota in healthy subjects, 35 middle-aged, non-obese Japanese people who are not currently treated with gastrointestinal disorders and not using sleep-inducing agents within last one month were involved in this study. The American Academy of Sleep Medicine and Sleep Research Society published consensus statement that healthy adults are recommended to sleep at least 7 h per night, and short sleep less than 7 h per night is associated with higher risk of diseases such as obesity, diabetes, hypertension, cardiovascular diseases, and depression.^54^ In this study, we revealed that shorter sleep time is associated with lower HD5 secretion into the intestinal lumen. In all the correlation analysis, we used Pearson’s correlation coefficient test because this method does not assume the normality of data.^55^ Sleep is closely associated with our immune functions, and short sleep relates to abnormal immune regulations such as higher concentrations of inflammatory markers such as IL-6 and CRP, increased number of white blood cells, decreased number of naïve T cells, lower NK cell activities, and also increased risk of infectious diseases.^3^ The intestinal epithelial cells play important roles in not only absorption of nutrients, but also immunity against the intestinal microbiota.^21^ Using animal models, it has been reported that sleep restriction decreases the expression of tight-junction proteins in colonic tissue^56^ and increases apoptosis of small intestinal epithelial cells,^57^ suggesting that short sleep impairs the integrity of intestinal epithelium. However, whether short sleep affects the immunological functions of the intestinal epithelium remains unclear. This study revealed that short sleep is associated with reduction of α-defensin secretion, one measure of innate enteric immunity by Paneth cells in the small intestine, providing novel insights into the relationship between sleep and gut mucosal immunity. We did not address precise mechanisms that short sleep relates to decrease of HD5 secretion from Paneth cells in this study, though, circadian rhythm may be involved. Expression level of mouse α-defensin Crps in wild-type mice shows circadian oscillation, elevated in the dark and decreased in the light.^50^ In addition, it has been reported that functional knockdown of clock genes such as *Bmal1* or *Per1/2* regulating circadian rhythm impairs the Wnt secretion cycle of Paneth cells,^48^ and *Per1/2* mutant mice show decreased Paneth cell number compared to wild-type mice.^49^ Although further studies are necessary to clarify the precise mechanism, impairment of circadian rhythm associated with short sleep may induce deficiency of Paneth cell α-defensin secretion.

CLR-transformed abundance of *[Ruminococcus] gnavus group, Butyricicoccus* and Enterobacteriaceae; unassigned was positively correlated, and *Bacteroides* was negatively correlated with both sleep time and fecal HD5 concentration, indicating that lower HD5 secretion is associated with the compositional disturbance of the intestinal microbiota along with short sleep. *[Ruminococcus] gnavus group and Butyricicoccus* also retained the correlation with sleep and HD5 concentration in the analysis of relative abundance with no CLR-transformation (data not shown), indicating an association among sleep, HD5, and the microbiome at the community level. In this study, whether the lower HD5 secretion causes the compositional disturbance of the intestinal microbiota was unclear. However, because properly folded α-defensins selectively kill pathogenic bacteria, whereas show no or minimal bactericidal activities against commensal bacteria^29^ and HD5 transgenic mice show different composition in the small intestinal microbiota compared to wild-type mice^30^, secreted Paneth cell α-defensins regulate intestinal microbiota composition by eliciting selective bactericidal activities. Thus, it is suggested that lower HD5 secretion along with short sleep induces the change of these genera. *Ruminococcus gnavus* is a mucin-degrading bacteria belonging to Clostridia cluster XIVa,^52^ and has ability to produce acetate from degradation products of starch released by other bacteria.^58^ Recent study reported that oral administration of *Ruminococcus gnavus* to atopic dermatitis model mice ameliorated dermatitis symptoms along with increase of regulatory T cell in skin and mesenteric lymph node and butyrate concentration in cecum,^59^ suggesting anti-inflammatory effects via modulating SCFA production in the intestine. *Butyricicoccus* is known as a butyrate-producing genus in the intestine.^53^ *Butyricicoccus pullicaecorum*, one of the species belonging to this genus, shows anti-inflammatory effects both *in vitro* and *in vivo*. ^60^ Furthermore, patients with Parkinson’s disease known that short sleep is a risk factor show lower occupancy of *Butyricicoccus* compared to healthy controls.^61^ In addition, it has been reported that Enterobacteriaceae contribute to protection against the colonization of harmful bacteria in the intestine^62,63^ and higher occupancy of *Bacteroides* relates to sleep disorders such as acute insomnia, obstructive sleep apnea syndrome, and short sleep.^64–66^ Taken together, lower HD5 secretion along with short sleep may be associated with increased risk of various diseases through the compositional disturbance of the intestinal microbiota.

Moreover, fecal SCFA concentration was positively correlated with sleep time and fecal HD5 concentration, suggesting that lower HD5 secretion along with short sleep is associated with decreased SCFA production by the intestinal microbiota. SCFAs produced by the intestinal microbiota contribute to the regulation of many aspects of host physiological functions. These include promotion of growth and barrier function of the intestinal epithelium, anti-inflammatory function via inducing regulatory T cell differentiation and inhibition of inflammatory cytokine production by macrophage, glucose metabolism regulation via promoting GLP-1 secretion by enteroendocrine cell and differentiation of β cell in pancreas, and promotion of nervous cell development in hippocampus.^67–70^ It has been further reported that fecal SCFA concentration is low in patients with diseases such as inflammatory bowel diseases,^71^ diabetes,^72^ and Parkinson’s disease.^73^ Taken together, lower HD5 secretion along with short sleep is suggested to relate with increased risk of various diseases through lower SCFA production by the intestinal bacteria. SCFAs are also known to relate to sleep regulation. In animal models, SCFAs produced by the intestinal microbiota regulate the expression of clock genes in the host,^74^ and butyrate administration induces sleep.^75^ Furthermore, lower intake of dietary fiber, which is a substrate of SCFA production by the intestinal microbiota in healthy subjects is associated with shorter sleep time^76^ and lower rate of slow-wave sleep known as deep sleep stage.^77^ SCFA receptors *GRP41* and *GRP43* are expressed in Paneth cells, and butyrate induces α-defensin secretion from Paneth cells.^27^ Thus, decreased SCFA production related to lower HD5 secretion along with short sleep may be associated with malignant cycles in progression of abnormal HD5 secretion and sleep disorders.

Involvement of the intestinal microbiota in regulating the brain-gut axis, which is the interaction between brain and intestinal functions, has been known. Relationships between decrease of SCFAs in the intestinal lumen and brain diseases such as anorexia nervosa, Parkinson’s disease, and autism spectrum disorder have been reported,^78^ indicating that the intestinal microbiota is an important player in regulating interaction between the brain and intestine. A recent study suggested the involvement of decreased α-defensin secretion in depression by disrupting brain-gut axis.^41^ In this study, we revealed the relationship between lower HD5 secretion along with short sleep and disturbance of the intestinal microbiota accompanied by lower SCFA production. Our findings highlight that Paneth cell α-defensin may contribute to the regulation of human brain-gut axis.

Recent sleep deprivation studies both in human^19^ and mouse^20^ have shown that short sleep induces dysbiosis. On the other hand, mice depleted the intestinal microbiota by antibiotic administration showed abnormal sleep cycle with increased time of rapid eye movement (REM) sleep in the light which is resting phase for mice and decreased time of both REM and non-REM sleep in the dark which is active phase,^79^ suggesting that dysbiosis induces abnormal sleep and intervention to the intestinal microbiota may improve sleep disorders. It has been reported that qualitative or quantitative abnormalities of Paneth cell α-defensins are associated with pathological progression of dysbiosis-related diseases such as Crohn’s disease,^34–36^ obesity,^37^ GVHD,^38,39^ alcoholic steatohepatitis,^40^ and depression.^41^ Furthermore, oral administration of α-defensins improves GVHD,^80^ obesity,^81^ and alcoholic steatohepatitis^40^ along with recovery of the intestinal microbiota homeostasis. Our findings have a significant scientific value to show the correlation between sleep and the intestinal microbiota controlled by α-defensins, and further provide a novel insight into developing the therapeutics of sleep disorders by intervention of the intestinal microbiota.

This study has several limitations such as a relatively small sample size and singular subjective measure of sleep which does not always align well with objective measurements. In addition, this study does not address causal relationships among sleep time, intestinal microbiota, and HD5 secretion. Future studies targeting to different populations such as other age groups and patients with sleep disorders or sleep-related diseases and experimental sleep restriction will further strengthen the understanding about involvement of HD5 in the sleep regulation via brain-gut axis.

## Materials and methods

### Study design and population

All data and samples used in this study were obtained as part of the DOSANCO Health Study, a community-based study targeting residents in Suttu town, Hokkaido, Japan.^51^ Briefly, total 2,100 participants (977 males and 1,123 females) who were three-year-old or older and not living in nursing homes participated in the study and responded to a self-administered questionnaire about their age, gender, medical history, and lifestyle. If participants were elementary school age or under, their parents answered the questionnaire instead.

In this questionnaire, the average sleep time per day was obtained by a free description in minutes based on the response to the following question: “*During the past month, how many hours of actual sleep did you get at night on average per day? (This may be different than the number of hours you spent in bed.)”*.

Six hundred twenty-nine of 2,100 participants consented to provide fecal samples. Fecal samples were collected by participants themselves using collection kits distributed beforehand, packed into a cooler bag with frozen refrigerants, and directly brought to the researchers in Suttu town and immediately stored at −30°C. Then, fecal samples were sent to the laboratory in Hokkaido University with dry ice and immediately stored at −80°C after arrival at the laboratory. Of these 629 fecal samples, 331 with enough amounts were subject to further analyses. From these 331 participants, 296 participants were excluded due to undergoing clinical treatment for diabetes, gastric ulcer, duodenal ulcer, hepatitis, liver cirrhosis, and other digestive system diseases which may directly influence the intestinal environment (*n* = 74), insufficient data quality of 16S rDNA sequencing (*n* = 61, detailed exclusion criteria was mentioned in 16S rDNA-based taxonomic analysis section), use of sleep medicines in the past month of the survey (*n* = 38), outside range of body mass index (BMI) from normal weight or overweight (18.5 ≤ BMI < 30 kg/m^2^) based on WHO criteria^82^ which may be at potential disease risk (*n* = 19), and outside range of age from the area of interest in this study (41 to 60-year-old) (*n* = 104). Finally, data from 35 participants including 15 men and 20 women were analyzed. This study was approved by the Ethical Committee of the Faculty of Medicine (15–002, 15–045), Hokkaido University, and written informed consent was obtained from all participants.

### Quantification of fecal HD5 by sandwich ELISA

Fecal samples were lyophilized and pulverized to powder using a beads-beater type homogenizer (PV1001, Yasui Kikai, Corp., Osaka, Japan). Ten mg of fecal powder was suspended with 100 µL of PBS (-), vortex-mixed overnight at 4°C, and centrifuged at 15,000 g for 30 min at 4°C. Then, supernatants were subjected to measurement of HD5 by sandwich ELISA established previously.^42^

### 16S ribosomal DNA (rDNA) sequencing

Total genomic DNA was extracted and purified from fecal samples, then, amplicon 16S rDNA library was constructed and sequenced on a MiSeq instrument (SY-410-1003, illumina, Inc., Hayward, CA) as previously described.^42^

### 16S rDNA-based taxonomic analysis

Demultiplexed pair-end fastq files obtained from MiSeq were analyzed by QIIME2 pipeline (version 2019.7).^83^ Sequences were quality-filtered, denoised, and chimera removed by DADA2 plugin^84^ with following parameters: –p-trim-left-f 17; –p-trim-left-r 21; –p-trunc-len-f 280; –p-trunc-len-r 200; –p-max-ee-f 2; –p-max-ee-r 2. After this step, samples that percentage of chimeric sequences exceeded 50% were excluded because sequence data quality was considered insufficient for the analysis. Then, phylogenic tree was created by FastTree^85^ after alignment with MAFFT.^86^ Taxonomy of each feature were assigned based on 99% sequence similarities to the Silva database (v132). To calculate the relative abundance of each genus, the number of sequencing reads was rarefied to 5,000 per sample. Also, count number of a taxon of interest was CLR-transformed. Correlation analysis between sleep time and CLR-transformed abundance of each genus is performed separately.

### Quantification of fecal SCFAs by high-performance liquid chromatography

A part of each fecal sample was sent to the contract analysis (Technosuruga Laboratory Co. Ltd., Shizuoka, Japan). Then, fecal concentrations of acetate, butyrate, and propionate were measured using a high-performance liquid chromatography system as described previously.^87^ Fecal concentrations of SCFAs were expressed in mg/g of fecal dry weight.

### Statistical analysis

All statistical analyses were conducted by GraphPad Prism ver. 9.0 software (GraphPad Software Inc., San Diego, CA). Pearson’s correlation coefficients test was used for statistical analyses. In all statistical tests, *p* < 0.05 was considered as statistically significant.

## Data Availability

The data that support the findings of this study are available on request from the corresponding author, Kiminori N. The data are not publicly available due to their containing information that could compromise the privacy of research participants.
